# A study to identify the practices of the buffalo keepers which inadvertently lead to the spread of brucellosis in Delhi

**DOI:** 10.1186/s12917-018-1670-2

**Published:** 2018-11-06

**Authors:** Nimita Kant, Parul Kulshreshtha, Rashmi Singh, Anuradha Mal, Amita Dwivedi, Riya Ahuja, Rinkle Mehra, Mohit Tehlan, Paritosh Ahmed, Shilpa Kaushik, Shipra, Shashikant Kumar, Aas Mohammad, Shrikrishn Shukla, Damini Singh, Rakesh Bhatnagar

**Affiliations:** 1 Department of Zoology, Shivaji College, University of Delhi, 110027, New Delhi, India; 2 Department of Botany, Shivaji College, University of Delhi, 110027, New Delhi, India; 3 School of Biotechnology, Jawaharlal Nehru University, 110057, Delhi, India; 4 Laboratory of Molecular Biology and Genetic Engineering, School of Biotechnology, Jawaharlal Nehru University, New Delhi, 110067 India

**Keywords:** Brucella, Delhi, Cattle keepers, Practices, Habits

## Abstract

**Background:**

India has the largest Buffalo population in the world, with every household in rural India owning buffaloes depending upon daily milk requirement – dairy farmers can own between 10 to 70 buffaloes. The health of Indian buffaloes is of economic importance since India is one of the largest buffalo meat exporters in the world, and Indian Buffalo semen is sold in the USA for breeding purposes. However, National Control Program on brucellosis is only active in South India and in Panjab (a North Indian state with high human brucellosis incidence). Our aim was to assess the knowledge and practices of the buffalo keepers of Delhi that make them susceptible to brucellosis.

**Results:**

Amongst all the 11 districts of Delhi, there was 0% awareness about brucellosis and also about the S19 vaccine as the buffalo keepers had never heard of S19 vaccine which is available at minimal cost from Indian Veterinary Research Institute, Bareilly, India. Majority of the respondents drink raw milk, sleep in cattle sheds, do not isolate sick cattle, do not test buffaloes blood for any disease before purchasing them, apply intrauterine medication with bare hands to buffalo after abortion of foetus, never clean their cattle sheds with a disinfectant and believe that they can only acquire skin infections from cattle. All of these habits make them prone to brucellosis. While about 20 to 27% of respondents reported a history of abortions and retained placenta, disposed of the placenta with bare hands, and applied raw milk on cracked lips. It was surprising to note that majority of them never reared small ruminants like sheep and goat with buffaloes or *Bos* species as they were aware of the rapid spread of disease from small to big ruminants.

**Conclusions:**

We found that buffalo keepers were ignorant of brucellosis, its causative agent, relevant vaccines and that they also involved in high-risk activities. As such, our findings highlight a need for buffalo keepers to be better educated via several awareness camps to minimize human exposure to *Brucella* in Delhi.

**Electronic supplementary material:**

The online version of this article (10.1186/s12917-018-1670-2) contains supplementary material, which is available to authorized users.

## Background

Brucellosis is a classified group III risk disease which has easy airborne transmission [6, 42]. *Brucella*, the causative agent of brucellosis is classified by Centers For Disease Control and Prevention or CDC as a category B pathogen that has the potential to be developed into a bioweapon. Brucellosis is endemic in India and affects dairy farming [19, 33, 40]. Bovine brucellosis has been discovered as the main reason for disease propagation in humans due to animal handling and consumption of bovine products [11]. Countries like Australia, Canada, Cyprus, Japan, Denmark, Finland, The Netherlands, New Zealand, Norway, Sweden and the United Kingdom have been able to eradicate human brucellosis because they have eradicated bovine brucellosis [7, 26]. Although some reliable reports are available from Western countries, brucellosis is always underreported in Asian countries. It has been identified that there is lack of data related to brucellosis from India, China and Sub-Saharan Africa [29].

The world organization for animal health (OIE) defines standards for surveillance, diagnosis, epidemiology, control, eradication efforts, and the reduction of risk for animal health [16]. It means that all these factors must amalgamate to establish a better healthcare system. India has been identified as one of the hotspots for emerging infectious diseases and brucellosis is one of the emerging diseases [38]. New Delhi, the National Capital Territory of India, boasts of one of the best health care system in India. But as yet the website of the Development department of Delhi Government [http://delhi.gov.in/wps/wcm/connect/lib_development/Development/Home/Citizen+Charter] does not give any information about brucellosis. The diagnostic innovations are of no use if people of the country have no knowledge about the disease. Therefore, spreading awareness is the most important aspect of a control program. Although the brucellosis control program is very aggressive in South India [14, 28], but it is very frail in North India except in Punjab which is a North Indian state with a high incidence of brucellosis [5]. During our study, we observed that the majority of the dairy farmers reared exclusively buffaloes in Delhi. Many among them reared *Bos* species in fewer numbers (to fulfil the household requirement) than buffaloes on the same farm. The reason for this practice was explained to be the thicker milk quality along with the higher milk volumes derived from the buffaloes. As the spread of infectious diseases can be regulated by amending the practices and taking precautions thus we did a survey to analyze the practices of the buffalo keepers that may be furnishing the spread of brucellosis from the infected buffaloes. At the same time, we informed the buffalo keepers of the ways to rectify their animal handling so as to improve animal health and to curtail the spread of brucellosis.

## Methods

### Informed consent

All the participants signed a consent form prior to responding to the survey. The questionnaires were signed by the cattle keepers after filling them.

### Sampling

A purposive sampling of the buffalo keepers was conducted from August 2015 to December 2016 in order to analyze their practices which promote the spread of brucellosis in the National Capital Territory of New Delhi, the capital city of India. As brucellosis is regarded as an occupational hazard, therefore, this study was conducted by interviewing the buffalo keepers of different districts of Delhi. The buffalo keepers come in close contact with the livestock therefore they constitute the high risk population for brucellosis. The buffalo keepers here refer to the human population of Delhi who reared buffalo exclusively for their household needs or for dairy farming. This human population also includes those who reared few *Bos* species on their cattle sheds to fulfil their household demands or for a few customers. This survey does not include data from the cattle keepers who exclusively reared domesticated cattle (*Bos* species) only. According to the census of 2012 there are 162,142 female and 20,445 male buffalos in rural and urban area of New Delhi. This includes the population of 11 districts of Delhi. The current census report is pending. We surveyed 1200 cattle sheds which included data of 5550 buffaloes (4828 females and 722 males) only. It is pin-pointed that the *Bos* species (usually 1 to 3 in number on each farm) on the surveyed cattle sheds were not included in this animal count. There is no report on the exact number of cattle sheds housing buffaloes per district in Delhi. Here, a ‘cattle shed’ is defined as the cattle establishment used to house buffaloes only or to house a few *Bos* species with buffaloes. These cattle establishments varied in the structure being close-house or open-house or some completely on the road-side or in the colony lanes between boundary walls of houses. The pictures of various cattle sheds are available as Additional files 1, 2, 3, 4 and 5. They do not resemble any buffalo farm advertised online by Indian companies as the common man cannot afford them. We tried to tap the maximum number of cattle sheds in each district but many people were not ready to interact. Therefore our study pertains to only those buffalo keepers who were ready to interact with the college students.

### Questionnaire

A structured questionnaire was developed in English language and translated in the Hindi language (the native tongue of North Indians). The questionnaire contained both open-ended and close-ended questions. The face to face method of approach was employed to collect the data. The questionnaire is available as the supporting file. The questionnaire revolved around the issues affecting the spread of brucellosis like biosecurity, reproductive health of the livestock, maintenance of cattle sheds and knowledge about zoonoses. The issues addressed by the questionnaire and their relevance to brucellosis have been discussed in Table 1. Each buffalo keeper was asked the number of male and female buffalo housed in their cattle sheds, they were asked if they got their cattle vaccinated or not, if yes then they were asked to name the vaccines/ medicines they injected their cattle with. Each buffalo keeper was asked if their buffaloes were prone to miscarriages, if yes then in which month; they were asked if they separated sick animal from the healthy ones; they were also asked if they consumed unboiled/ unpasteurized milk; accessibility to veterinary doctors was also asked; buffalo keepers were asked how often do the Government Organizations come for blood tests. They were asked about the precautions they took while handling an aborted fetus. They were asked if the adult female buffaloes were milked; socio-economic data, medical histories. In order to receive an unbiased response, the disease of interest was not revealed to the respondents and the question regarding their knowledge about brucellosis was only asked at the end. On the completion of the questionnaire, the respondents were informed about brucellosis, safe livestock handling, S19 vaccinations and other measures to prevent the spread of brucellosis. Data validation was done during data collection in the field and also at the time of translation to English. Responses to the close-ended questions have been tabulated and responses to open-ended questions have been elaborated in result and discussion section. Data from questionnaires were entered in Microsoft Excel2010. “Yes” or “No” or “don’t know” responses were recorded as per the response for each respondent. The ‘countif’ application was used to find the percentage of each response for each question.

**Table 1:** Questionnaire pertained to the matter of biosecurity, reproductive health of the livestock, maintenance of cattle sheds and knowledge of zoonoses

S.N.	Issue addressed	Relevance to Brucellosis
1.	Consumption of raw milk and Application of raw milk on cracked lips	Infected buffalo secrete large amounts of *Brucella* in their milk.
2.	Assisting animal birth, application of intrauterine medication post abortion, disposing aborted foetus and placenta with naked hands.	Uterine fluid, Placental membranes, aborted foetus of infected buffalo during parturition or abortion are rich sources of *Brucella.*
3.	History of abortion and retained placenta	The seroprevalence of brucellosis is found to be significantly higher in animals with a history of abortion and retained placenta.
4.	Knowledge about Brucellosis or any other zoonoses and S19 vaccine	Knowledge about a disease makes the high risk population cautious and thus prevents the spread. Vaccination of young animals is known to reduce burden of disease.
5.	Rearing small ruminants with large ruminants	Infectious diseases spillover from small ruminants to large ruminants and cause huge economic loss.
6.	Sleeping in cattle sheds	Close contact with buffalo is a risk factor identified for human Brucellosis.
7.	Blood testing before sale and purchase of cattle	Diagnosis of brucellosis may curb the sale of non-productive buffalo and curb the spread of disease.
8.	Separation of sick animals	Intermingling of sick buffalo or domesticated cattle like *Bos* sp. with healthy buffalo may facilitate the transmission of brucellosis to susceptible cattle.
9.	Use of disinfectant to clean the cattle shed	Disinfectants lyse the gram negative bacteria and thus remove infection from the environment of the cattle shed.

## Results

### Income groups

**Fig. 1:**
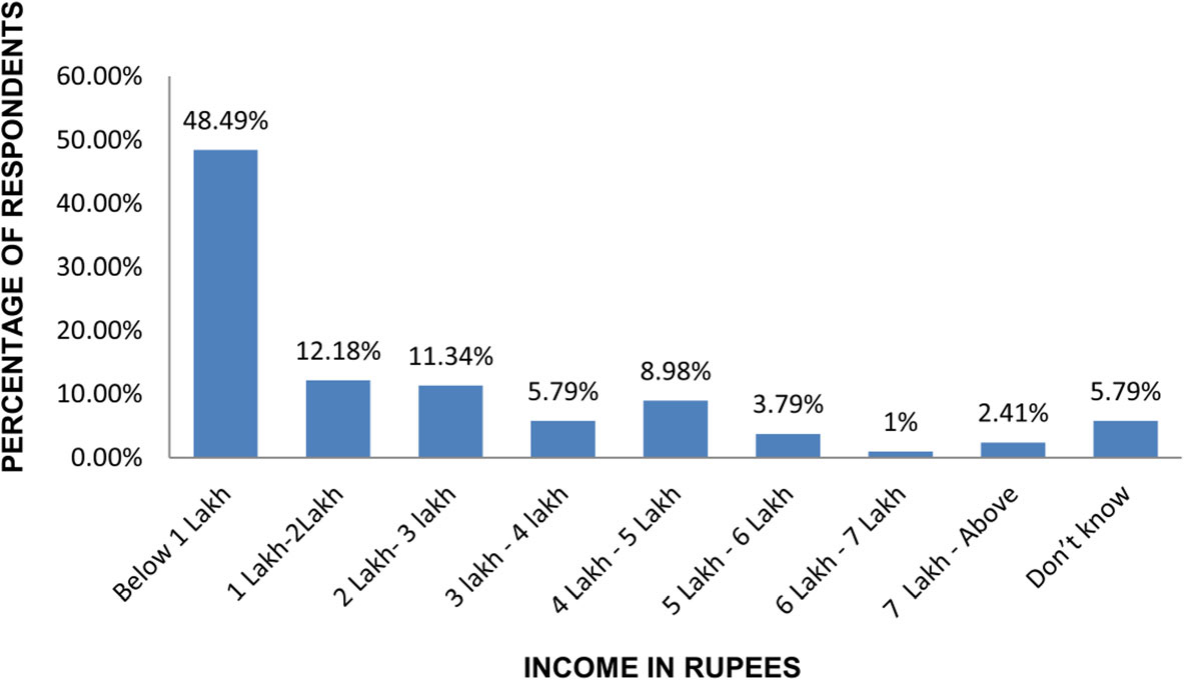
Annual income of the respondents in Indian currency (Rupees)

As brucellosis is linked to the economic status of people, therefore, we enquired about the income of each respondent from districts tabulated in Table 2. Cattle sheds surveyed in New Delhi, Central Delhi, West Delhi and South East Delhi were fewer as these areas fall in the urban area with fewer cattle establishments. As is evident from Fig. 1, it can be seen that the 50% of the respondents belonged to the least income group of less than Rupees one Lac per annum, while rest of the respondents fell into higher slabs. It is reiterated here that 1 Lac rupees are equivalent to 1500 US$. It can be seen that a meagre percentage of 2.41% of respondents belonged to the category of people who earned more than 7 Lac rupees per annum. About 5.79% did not know their income. Thus it can be concluded that most people belonged to the lower income group in our survey.

**Table 2:** The survey rate of the buffalo keepers per district of Delhi, India

S.N.	District	Headquarter	Subdivisions	Number of cattlesheds surveyed
1	New Delhi	Connaught Place	Chanakyapuri, Delhi Cantonment, Vasant Vihar	20
2	North Delhi	Narela	Model Town, Narela, Alipur	164
3	North West Delhi	Kanjhawala	Rohini, Kanjhawala, Sarawati Vihar	150
4	West Delhi	Rajouri Garden	Patel Nagar, Panjabi Bagh, Rajouri Garden	85
5	South West Delhi	Dwarka	Dwarka, Najafgarh, Kapashera	171
6	South Delhi	Saket	Saket, Hauz Khas, Mehrauli	128
7	South East Delhi	Defence Colony	Defense Colony, Kalkaji, Sarita Vihar	34
8	Central Delhi	Daryaganj	Kotwali, Civil lines, Karol Bagh	42
9	North East Delhi	Seelampur	Seelampur, Yamuna Vihar, Karawal Nagar	128
10	Shahdara	Shahdara	Shahdara, Seemapuri, Vivek Vihar	150
11	East Delhi	Preet Vihar	Preet Vihar, Gandhi Nagar, Mayur Vihar	128

### Knowledge about brucellosis

As is evident from Table 3, 0% of respondents knew about brucellosis. Thus they were not cognizant of *Brucella* infections. In our study, it was observed that 37% of cattle keepers got their cattle vaccinated but only 9.25% of respondents could name any vaccine. The vaccines named by these respondents included anthrax vaccine, Foot and Mouth Disease or FMD vaccine, Enterotoxaemia or ET and Black Quarter or BQ vaccine. None of the respondents could name S19 or RB51 vaccine. Exactly 13% of respondents expressed that they do not know if their cattle is vaccinated or not. While 15% of respondents agreed that they only sometimes get their cattle vaccinated. Therefore it could be concluded that awareness about brucellosis was completely absent amongst the buffalo keepers. Majority of respondents in our study, 98%, conveyed that they did not rear small ruminants with buffaloes because when a disease affects one small ruminant then it spreads to all the big and small ruminants in the cattle sheds resulting in a huge loss. Thus it can be said that the risk of *Brucella* infection spilling from small ruminants to large ruminants was not significant amongst the population surveyed. Thus the buffalo keepers are conscious of the danger of the rapid spread of infection from small to large ruminants. It was observed that many buffalo keepers of Delhi kept dogs on the cattle shed as an alarm system to alert the owner of cattle thieves. Most cattle sheds were open house thus there is a high probability of the contamination of the feed and water by infected stray dogs also. Dogs in the cattle shed form a parallel reservoir of *Brucella* because dogs shed *Brucella* in reproductive fluids and spread bovine brucellosis [31]. Despite its endemism it was confounding to observe ignorance towards brucellosis, thus during our study, we apprised the buffalo keepers about brucellosis and S19 vaccination in detail.

**Table 3:** Knowledge and Practices of the buffalo keepers of Delhi, India

S.N.	Risk Factors	Yes %	No %	Sometimes %	Don’t Know %
1	Drink raw milk	38	59	3	0
2	Milk the animal	60	35	5	0
3	Sleeping in Animal Sheds	79	11	10	0
4	Assisting Animal Birth	100	0	0	0
5	History of abortion in [3rd trimester] on farm	21	79	0	0
6	Disposed aborted fetus with naked hands	24	76	0	0
7	Incidence of retained placenta	20	70	10	0
8	Disposed placenta with naked hands	27	73	0	0
9	Applying raw milk on cracked lips	22	71	7	0
10	Vaccination of animals	37	35	15	13
11	Isolation of sick animals	32	68	0	0
12	Can you acquire disease from your cattle	37	38	25	0
13	Applied Intrauterine medication with naked hands after abortion	45	50	5	0
14	Use of disinfectant to clean cattle shed	13	85	2	0
15	Blood test before buying the animal	0	100	0	0
16	Do you rear goat and sheep with Buffalo and cow?	0	98	2	0
17	Have you heard of Brucellosis?	0	100	0	0

### Availability of the veterinary services

In an open-ended question, all the respondents were asked to summarize the state of the veterinary services available to them. We were dismayed to know that the veterinary services in all the districts of Delhi were extremely poor. All respondents revealed that there have never been any awareness camps regarding brucellosis in their area. It is reiterated that the website of the Development department of Delhi Government does not give any information about brucellosis. Respondents of our study from remote villages of South West Delhi divulged that the sweepers of government hospitals learn to deliver intravenous injections. Such sweepers act as veterinary doctors and visit the village in hope of mining money. Respondents also shared that these sweepers inject tetanus toxoid for every health problem and charge Rs.700 per buffalo. Thus, the respondents shared that in case of ill health they inject the buffaloes with antibiotics like terramycin by themselves without any veterinary intervention (Additional file 6). They also informed that they take the buffaloes to the hospital only during worst case scenario.

### Practices

To assess if the practices of buffalo keepers increase the risk of *Brucella* infection, a number of questions were included in the questionnaire. It was found that 38% of respondents drank raw milk. These respondents agreed that they sometimes drank directly from the udders of cow or buffalo because they considered lactating animal as their religious mother. The practice of drinking raw milk driven by this belief puts these respondents at a risk of acquiring brucellosis. Respondents (65%) were also involved in milking the animal, 5% of respondents expressed that they sometimes milk the animal. As this activity involves touching the udders and coming in contact with raw milk thus this puts a large number of respondents at risk of acquiring brucellosis infection. Respondents from all the districts of Delhi revealed that their cattle sheds were prone to the theft of buffalo. Therefore, 79% of respondents always sleep in cattle sheds while 10% agreed that they only sometimes sleep in cattle sheds. *Brucella* can survive in soil for two to 6 months, therefore, sleeping in cattle sheds increases the probability of air-borne transmission or infection via an abrasion in the skin of buffalo keepers [23, 24]. All the respondents assisted the animal birth as it is celebrated as a special event in their family. Abortion in the 3rd trimester is a characteristic of brucellosis, and it was noted that 21% of surveyed cattle had abortions during this time. Thus it could be concluded that such buffaloes had the symptoms of brucellosis. Post-abortion the buffaloes are given tetanus toxoid intravenously only and no other medication was given. A low rate of abortion does not warrant a low prevalence rate as it is known that brucellosis may prevail as a silent disorder. Most buffalo keepers from Delhi confided that the buffaloes which suffered frequent abortions are sold off in local cattle fair. Thus such buffaloes stay in circulation as a major carrier of brucellosis. This practice will further deteriorate the state of affairs. We found that the aborted foetuses were disposed of with naked hands by only 24% of respondents as most of them call doctor under such situation. Cattle keepers in rural areas (22% of respondents) of Delhi used raw milk to heal cracked lips while in urban areas most cattle keepers (71% of respondents) applied commercially manufactured creams on cracked lips while 7% of respondents agreed that they applied raw milk on cracked lips only sometimes. Applying raw milk to heal cracked lips is an age-old traditional therapy in India which can also cause *Brucella* infection in humans. The incidence of retained placenta in buffaloes was reported by only 20% of respondents as 70% denied any retention and 10% expressed that this happened only sometimes. Isolation of sick animals was not being followed by 68% of respondents, thus these cattle keepers put their healthy cattle at a risk of the infectious diseases harboured by sick animals. Many respondents (37%) expressed that they could acquire some disease from their cattle, 25% of respondents said that they may sometimes acquire infection from cattle. These respondents suggested that the skin infections could be acquired from the cattle. Many respondents (38%) thought that they cannot acquire any infection from cattle. They also shared that even the sickness of the buffalo does not hinder them from consuming its milk. Drinking milk of a sick buffalo is a huge health hazard which cannot be avoided until the buffalo keepers are not convinced about the potential of disease communication from buffalo to them. At the end of the survey, we informed the buffalo keepers about the routes of disease communication from buffalo to humans.

Only 45% of respondents agreed that they applied intrauterine medication with naked hands post -abortion, 5% of respondents said that they only sometimes practiced this. While 50% of respondents expressed that they do not apply any medication post abortion. Application of intrauterine medication with naked hands post-abortion again is a practice which may communicate brucellosis from buffalo to the buffalo keepers. Assisting parturition exposes the buffalo keepers to fetal membranes, aborted fetus and uterine fluid contaminated with *Brucella* species [3, 17]. Only 13% of respondents expressed that they used disinfectant to clean the cattle shed, a meagre 2% agreed that they utilized disinfectant for this purpose. Exactly 85% of respondent agreed that they never used disinfectants to clean the cattle shed rather they washed the sheds with water and throw dry sand over the sheds to clean. In such an environment the propagation of several infectious diseases becomes most probable.

## Discussion

Brucellosis has not been listed amongst the neglected tropical disease in India and South East Asia [22], but it has been identified as a neglected tropical disease by WHO, the World Health Organization [43]. Brucellosis is known to cause huge economic losses in India ranging between a loss of US $ 6.8 per cattle and US$18.2 per buffalo [37]. Brucellosis has been listed amongst zoonosis that affects the health of poor and affects the trade of animal products [41]. In our study, most of the respondents hailed from a poor background, therefore, it was pertinent to assess the economic status of the respondents of our survey. India comprises a big geographical entity, consequently, the epidemiology of brucellosis varies from one region to another. Seroprevalence varies from 3.3–11.4% in Chennai only, while Isloor et al reported overall prevalence for Karnataka to be 1.9% in cattle and 1.8% in buffalo. The Indian Agricultural Research Institute reported a 13.5% of stable endemic equilibrium for brucellosis in India [15, 30, 36]. Rahman et al. recognized that Delhi has the highest seroprevalence but the exact data was not published [30]. The Project Directorate on Animal Disease Monitoring and Surveillance (PDMAS, India) under the Ministry of Agriculture launched “Vision 2030” in 2011 [30] to eradicate brucellosis from India by the year 2030. The obligatory prerequisite for the success of any control program is building awareness about the disease. According to our study, the buffalo keepers from Delhi were totally unaware of brucellosis. The same level of awareness was reported from Kenya in the year 2007 and recently from Tajikistan [18, 21]. As opposed to this, the cattle keepers and shepherds from Egypt declared that their animals have a history of *Brucella* infection. They also confirmed that this infection was the main cause of abortions in their animals [10, 35]. Recent reports from Kenya show better awareness among the youngsters than the elderly. Better levels of awareness in recent years have been linked to higher seroprevalence of brucellosis in Kenya. Recent studies from other countries like Egypt, Tajikistan, and Kenya have reported that the livestock keepers were aware that brucellosis can spread from livestock to humans and that arthritis was a common symptom of the same [13, 21, 27]. In contrast to this, we found that cattle keepers from Delhi still think that only skin diseases can be acquired by handling cattle.

In western countries, there is a system of surveillance for brucellosis. Detailed data on brucellosis is compiled from time to time on demographics, the onset of symptoms, clinical signs, contact dates with the treating physicians, hospitalization, death, laboratory diagnosis, bacterial species, geographic origin and possible vehicle of infection. Standardized questionnaires containing these questions are sent to local health departments for every reported case of brucellosis. The same system needs to be developed in India as well. In terms of availability of information, India is 65 years behind the western countries [4, 12, 16, 32, 34]. Several countries identify their failures in controlling brucellosis [2] and discuss better ways along with newer possibilities to pin it down. This kind of model needs to be adopted by India as well. Other countries like the Gambia that report low prevalence for brucellosis should also be looked upon as a paradigm [9].

According to our study, the veterinary services in the rural areas of Delhi were not appropriate. Thus the buffalo keepers like to inject drugs by themselves in buffalo. Only when the problem escalates they take buffaloes to the remote veterinary hospitals. Studies from other countries like Egypt and Tajikistan also report reluctance on the part of the livestock keepers to contact veterinarians [11, 21]. It is also known that local dairies of India owned by the cattle keepers sell unpasteurized milk only. There have been reports of sale and purchase of unpasteurized dairy products in Iran, Egypt, Tajikistan, Uzbekistan, and Yemen also. This practice has been regarded as a prime risk factor for spread of human brucellosis [1, 8, 11, 39]. It is believed that even a sporadic abortion must be linked to brucellosis [3, 24] and the animal undergoing abortion must be culled. Many countries like India have reported the lack of official culling of infected sheep, goats, and buffalos as the main cause of high *Brucella* seropositivity [11]. Livestock keepers from other countries have reported that they feed the aborted foetuses to dogs or throw it in the water canal which adds to the spread of brucellosis [7]. This treacherous practice exposes the entire ecosystem to brucellosis. This habit was not reported by the buffalo keepers from Delhi. Studies from other parts of the world also report that gloves and masks are not being utilized while handling aborted foetuses and while assisting parturition [11, 20, 21]. All these practices make the spread of brucellosis convenient and rampant.

Most of the underdeveloped countries across the globe face the same situation but a developed country like the United States of America (USA) has identified brucellosis as a prioritized zoonoses. Furthermore, they have made a road map to combat not only the zoonoses but also the infectious diseases by gauging their own capabilities on the scale of surveillance and availability of diagnostics. Despite being a developed country, the USA has revealed an insufficiency of diagnostic capability [25]. Though the focus of our survey was brucellosis, we can conclude that the knowledge about most infectious diseases was insufficient, also the practices of these cattle keepers put them at high risk of acquiring infectious diseases. Thus, like developed countries, India must adopt a holistic approach to combat zoonoses and other infectious diseases as a whole. Our study may be regarded as only an elementary research due to several limitations. Our major limitation is the sampling bias. There is no report on the total number of cattle sheds in all the districts of Delhi. Therefore, it is impossible to pinpoint the percentage of the cattle keepers from Delhi who harbour the same opinion or perform the same practices documented in this study. Though, we tried to overcome this limitation by interviewing as many buffalo keepers as were ready to interact with us. Another limitation of this study has been the summation of responses of cattle keepers from household cattle sheds, small cattle sheds, and large cattle sheds together. As the situation and configuration of these cattle sheds differ, therefore, this may also be having a confounding effect on the inference.

## Conclusion

In the NCT of Delhi, we found that the cattle keepers have never been surveyed for their opinions and practices regarding any infectious disease. On interviewing the cattle keepers we realized that they are sensitive towards the medical needs of their cattle but they do not have appropriate help. Our study indicates that the cattle keepers are oblivious to the practices which cause spillover infection of *brucella* from their cattle to them. It can be concluded from our survey that the cattle farmers are ignorant of brucellosis, its causative agent, the route of its transmission, its symptoms and vaccination. The emotional and religious belief of the cattle keepers further exposes them to the threat of brucellosis. The absence of culling, free cattle trade and absence of blood testing before buying the cattle makes the situation even gross. Thus we endeavoured to enlighten the cattle keepers with the harmful husbandry practices which endorse the spread of brucellosis. The major problem faced by the control programs includes opinions of people and it is important to mould these opinions if brucellosis is to be combated successfully. In the current scenario, it is pertinent that the Indian Government must organize awareness camps before brucellosis becomes a bigger menace.

## Additional files

Additional file 1: a) Open house cattle shed in North East Delhi b) and c) Open house cattle sheds of the South West Delhi district. (PPTX 816 kb)

Additional file 2: A road-side establishment of North Delhi district. (PNG 627 kb)

Additional file 3: One side open cattle shed used for housing buffalo and *Bos* species together. (PNG 561 kb)

Additional file 4: Close house cattle shed in Shahdara district, arrow shows the opening of the housing area. This cattle shed was completely dark with light going through this opening. (PNG 373 kb)

Additional file 5: Buffalo residing in the lane between houses in the South Delhi district. (PNG 324 kb)

Additional file 6: One of the medicines shown by the respondents was Terramycin, Injectable solution. (PNG 242 kb)

Additional file 7: Questionnaire. (DOCX 12 kb)

## Abbreviations

BQ: Black Quarter
CDC: Centers For Disease Control and Prevention
ET: Enterotoxaemia
FMD: Foot and Mouth Disease
OIE: Organization for animal health
PDMAS: Project Directorate on Animal Disease Monitoring and Surveillance
USA: United States of America
WHO: World Health Organization
