# Supplementary figures and images for: A study to identify the practices of the buffalo keepers which inadvertently lead to the spread of brucellosis in Delhi
Source: BMC Vet Res. 2018 Nov 6;14:329. doi: 10.1186/s12917-018-1670-2 (PMC6219203; doi:10.1186/s12917-018-1670-2)

## Slide 1
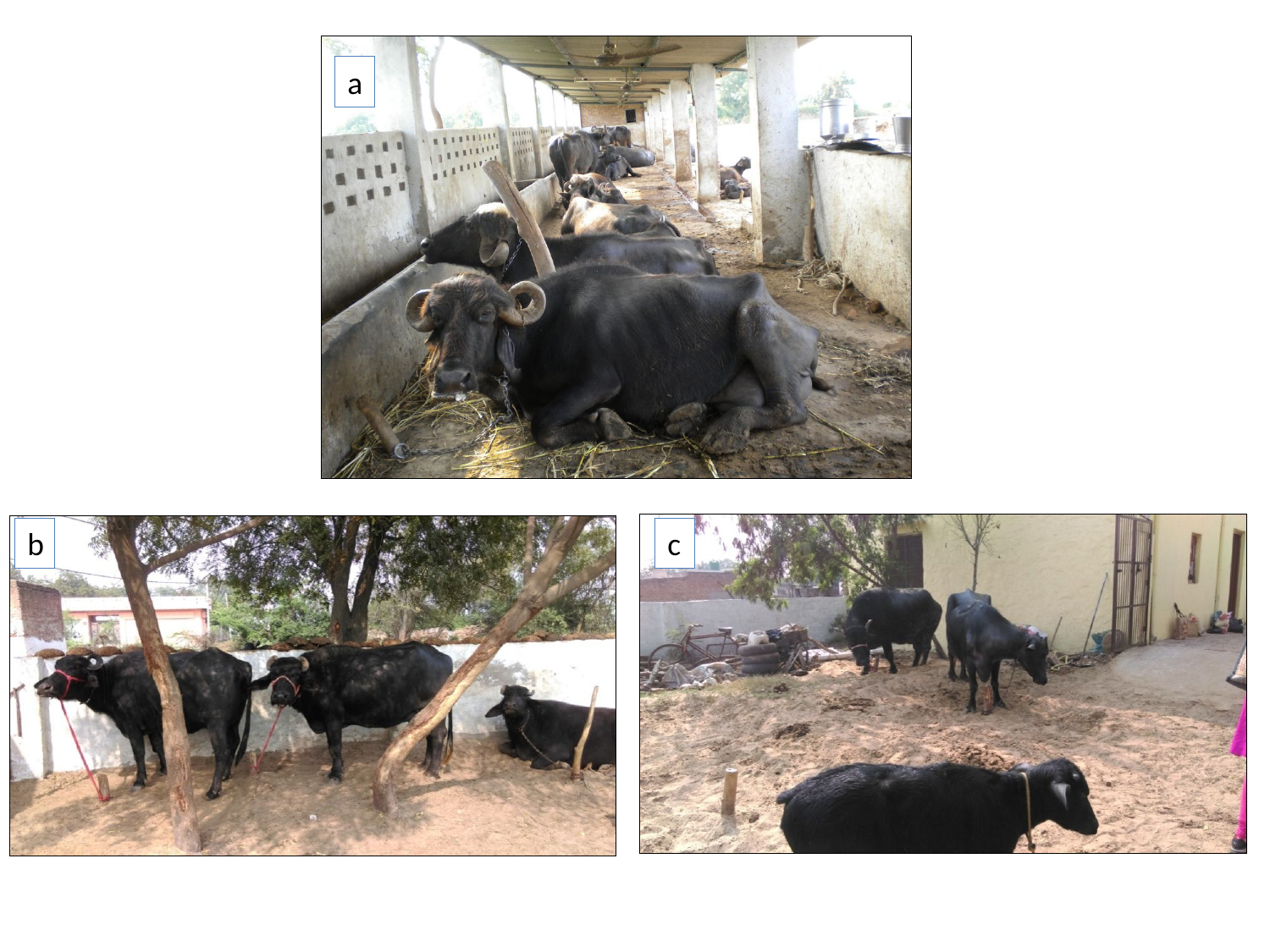

a
b
c

Supplement: Supplementary file 1 — a) Open house cattle shed in North East Delhi b) and c) Open house cattle sheds of the South West Delhi district. (PPTX 816 kb) [file 12917_2018_1670_MOESM1_ESM.pptx]

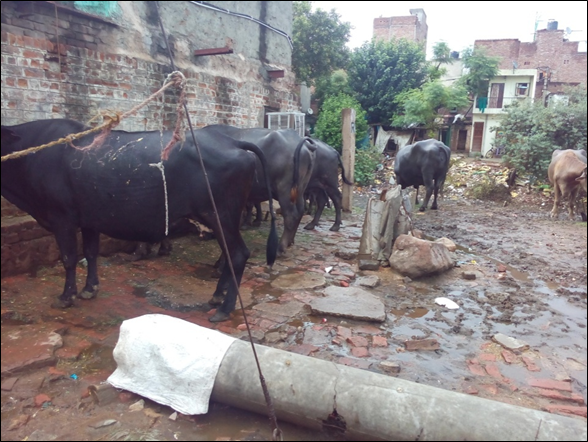

Supplement: Supplementary file 2 — A road-side establishment of North Delhi district. (PNG 627 kb) [file 12917_2018_1670_MOESM2_ESM.png]

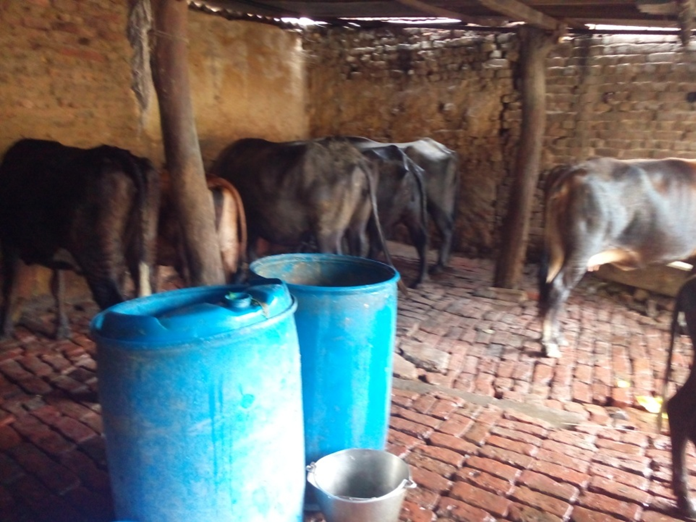

Supplement: Supplementary file 3 — One side open cattle shed used for housing buffalo and Bos species together. (PNG 561 kb) [file 12917_2018_1670_MOESM3_ESM.png]

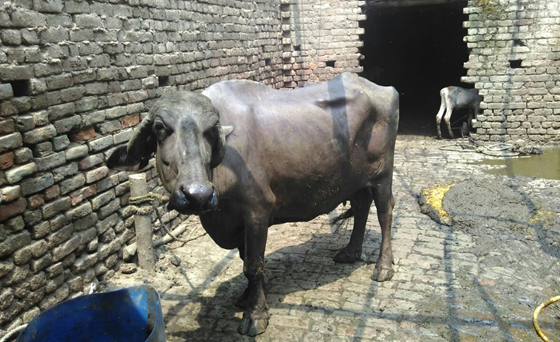

Supplement: Supplementary file 4 — Close house cattle shed in Shahdara district, arrow shows the opening of the housing area. This cattle shed was completely dark with light going through this opening. (PNG 373 kb) [file 12917_2018_1670_MOESM4_ESM.png]

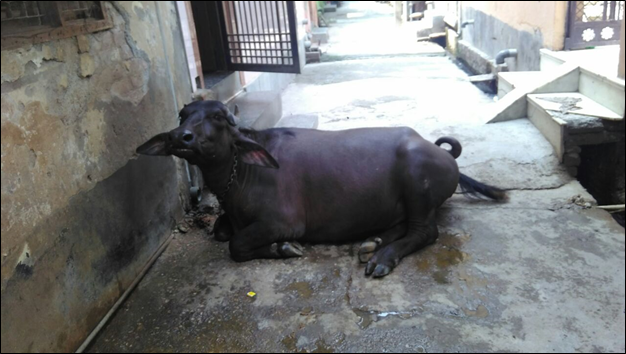

Supplement: Supplementary file 5 — Buffalo residing in the lane between houses in the South Delhi district. (PNG 324 kb) [file 12917_2018_1670_MOESM5_ESM.png]

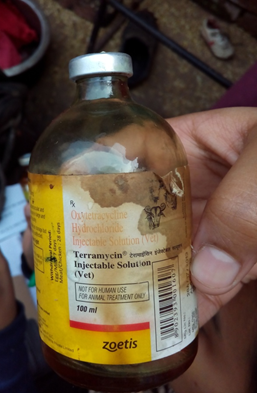

Supplement: Supplementary file 6 — One of the medicines shown by the respondents was Terramycin, Injectable solution. (PNG 242 kb) [file 12917_2018_1670_MOESM6_ESM.png]
