# Supplementary material for: A study to identify the practices of the buffalo keepers which inadvertently lead to the spread of brucellosis in Delhi
Source: BMC Vet Res. 2018 Nov 6;14:329. doi: 10.1186/s12917-018-1670-2 (PMC6219203; doi:10.1186/s12917-018-1670-2)
Supplement: Supplementary file 7 — Questionnaire. (DOCX 12 kb) [file 12917_2018_1670_MOESM7_ESM.docx]

Name:

Age:

Address:

Date:

Declaration

I agree to fill this questionnaire

Signature:

1. How many Buffalo you have?
2. How many are male/ female?
3. Does Government Veterinary officer/ Doctor visit your cattleshed and how often?
4. Have there been any awareness camps about cattle diseases in your area? If yes then how then how often do they occur?
5. Do you drink raw milk without boiling it?
6. Do you milk your buffalo?
7. Do you sleep in cattlesheds? If yes then provide the reason.
8. Have you assisted in cattle birth?
9. Do your cattle have a history of abortion? If yes then in which month?
10. Have you disposed aborted fetus by naked hand?
11. How many cases of retained placenta have you had on your farm?
12. Have you disposed retained placenta by hand?
13. Have you ever applied intrauterine medication with naked hands after abortion?
14. Do you apply raw milk on cracked lips?
15. Do you vaccinate your cattle? If yes then name the vaccines.
16. Do you isolate your sick animals?
17. Can you acquire disease from your animals? If yes then name the diseases.
18. How do you clean your cattleshed?
19. Do you use disinfectant to clean the cattleshed?
20. How do you select the buffalo before buying?
21. Do you conduct blood test to screen for any disease before buying the cattle?
22. Do you rear goat or sheep with cow or buffalo? If not then give reason.
23. Have you heard of Brucellosis?
24. Have you heard of S19 vaccine?
